# Metabolic Syndrome Prediction Models Using Machine Learning and Sasang Constitution Type

**DOI:** 10.1155/2021/8315047

**Published:** 2021-02-08

**Authors:** Ji-Eun Park, Sujeong Mun, Siwoo Lee

**Affiliations:** Future Medicine Division, Korea Institute of Oriental Medicine, Daejeon, Republic of Korea

## Abstract

**Background:**

Machine learning may be a useful tool for predicting metabolic syndrome (MetS), and previous studies also suggest that the risk of MetS differs according to Sasang constitution type. The present study investigated the development of MetS prediction models utilizing machine learning methods and whether the incorporation of Sasang constitution type could improve the performance of those prediction models.

**Methods:**

Participants visiting a medical center for a health check-up were recruited in 2005 and 2006. Six kinds of machine learning were utilized (K-nearest neighbor, naive Bayes, random forest, decision tree, multilayer perceptron, and support vector machine), as was conventional logistic regression. Machine learning-derived MetS prediction models with and without the incorporation of Sasang constitution type were compared to investigate whether the former would predict MetS with higher sensitivity. Age, sex, education level, marital status, body mass index, stress, physical activity, alcohol consumption, and smoking were included as potentially predictive factors.

**Results:**

A total of 750/2,871 participants had MetS. Among the six types of machine learning methods investigated, multiplayer perceptron and support vector machine exhibited the same performance as the conventional regression method, based on the areas under the receiver operating characteristic curves. The naive-Bayes method exhibited the highest sensitivity (0.49), which was higher than that of the conventional regression method (0.39). The incorporation of Sasang constitution type improved the sensitivity of all of the machine learning methods investigated except for the K-nearest neighbor method.

**Conclusion:**

Machine learning-derived models may be useful for MetS prediction, and the incorporation of Sasang constitution type may increase the sensitivity of such models.

## 1. Introduction

Metabolic syndrome (MetS) is a condition associated with multiple metabolic abnormalities, including obesity, hypertension, hyperglycemia, and hyperlipidemia [1]. The prevalence of MetS is reportedly increasing rapidly worldwide [2], and in South Korea, it rose from 22.6% in 2013 to 26.0% in 2017 [3]. MetS may be associated with various conditions such as cardiovascular disease [4] and cancer [5]. Therefore, it is important to diagnose MetS early and to manage risk factors in high-risk groups.

Previous studies investigating the prediction of MetS or attempting to identify associated factors have used statistical analysis methods such as linear regression or logistic regression [6, 7]. However, these methods sometimes have limitations, including strict assumptions or multicorrelation problems. In this sense, machine learning, a relatively new approach to modeling, may provide insight into MetS prediction. A number of recent studies investigating MetS prediction via machine learning-derived models have yielded promising results [8, 9].

In efforts to predict MetS accurately, several studies have incorporated Sasang constitution type as a potential risk factor. Sasang constitution type is based on traditional Korean medicine, and it classifies people into four types, Tae-Yang (TY), Tae-Eum (TE), So-Yang (SY), and or So-Eum (SE), based on physiological and pathological characteristics. Each type is associated with different disease susceptibilities, and in some previous studies, Sasang constitution type was associated with the risk of MetS [10, 11]. Specifically, the TE type was associated with a higher risk of MetS than the SY or SE types [10–12]. In addition, several studies suggested that each constitution type is differentially affected by different MetS risk factors [13].

To date, only a small number of studies have investigated the use of machine learning-based methods for MetS prediction, and the performances of the prediction models thus derived have varied considerably. No previous study has investigated the incorporation of Sasang constitution types into machine learning-based methods for MetS prediction. The aims of this study were (1) to develop MetS prediction models using machine learning methods and (2) to investigate whether Sasang constitution type improves the performance of the MetS prediction models.

## 2. Methods

### 2.1. Data Sources and Outcome Measurement

The current study used the Sasang constitution cohort data from the Korean Genome and Epidemiology Study (KoGES: 4851-302) [14]. The KoGES is a large cohort study project managed by the Korean National Institute of Health to investigate gene-environment factors in chronic diseases [15]. Those data include information derived from a total of 3,064 participants who received health check-ups during 2005 and 2006, and their data were collected only once. Therefore, the current investigation was a cross-sectional study.

During initial prediction model development, the potential predictors used were age, sex, education level, marital status, body mass index (BMI), stress, physical activity, alcohol consumption, and smoking. Demographic data including age, sex, marital status, education level, and employment status were assessed by trained investigators via a standardized questionnaire. Alcohol consumption, smoking, physical activity, stress, and BMI were measured one time and were analyzed in an effort to assess the effects of health-related behaviors. Alcohol consumption was calculated in g/day based on the frequency, amount per occasion, and the type of drinks. Physical activity was calculated in total metabolic equivalent-minutes per week considering the type of exercise, frequency, and time per exercise. Smoking status was classified as “current smoker,” “former smoker,” or “never smoked.” Stress was assessed via the Psychosocial Well-Being Index-Short Form [16]. That index consists of 18 items, and a higher score indicates greater psychosocial stress. BMI was calculated as weight divided by height (kg/m2). To diagnose MetS, blood pressure and waist circumference were analyzed, as were the results of blood tests including triglycerides, high-density lipoprotein, and fasting glucose. This study was approved by the Institutional Review Board of the Korea Institute of Oriental Medicine (I-1809/007-001).

MetS was diagnosed in accordance with the Adult Treatment Panel III [1]. Elevated waist circumference was defined as ≥90 cm for men and ≥85 cm for women. Elevated triglycerides were defined as ≥150 mg/dL, or being on drug treatment for elevated triglycerides. Reduced high-density lipoprotein cholesterol was defined as <40 mg/dL for men and <50 mg/dL for women, or being on drug treatment for reduced high-density lipoprotein cholesterol. Elevated blood pressure was defined as ≥130 mmHg systolic blood pressure or ≥85 mmHg diastolic blood pressure, or being on antihypertensive drug treatment. The blood pressure was measured using a mercury sphygmomanometer (Baumanometer®, W.A. Baum Co., Inc., Copiague, NY, USA) after participants rested in a seated position. After fasting at least eight hours overnight, participants underwent an early morning blood sample collection. Blood glucose was measured after fasting at least eight hours, and elevated fasting glucose was defined as ≥100 mg/dL or being on drug treatment for it. If any three or more of the above conditions were met, MetS was diagnosed.

Even though there are several questionnaires for Sasang constitution-type diagnosis such as the KS-15 [17] and SCAT [18], in the KoGES cohort study, Sasang constitution type was assessed by two independent Korean medicine doctors to increase the accuracy. One Sasang constitution medicine specialist made a diagnosis of the participants' constitution based on analysis of face, voice, and questionnaire (Appendix 1). After that, another specialist confirmed the diagnosis. If the diagnoses of the two specialists were different, it was resolved by discussion with a third specialist.

These analytic methods used in the process were developed in the College of Oriental Medicine of Kyung Hee University and validated [19–21]. For facial images, a digital camera was used to extract the facial characteristics such as width, height, areas, angle, depth, and forehead. The pitch, formant frequency, bandwidth, and other vocal parameters were measured for voice analysis. These voice features were processed using the Hidden Markov model toolkit and the Praat voice analysis program. In addition, for body shape, the circumference of the forehead, neck, axilla, chest, ribs, waist, pelvis, and hips was measured. The questionnaire included questions related to general temperature, eating habits, physiological symptoms, sweating, and temperament [13].

### 2.2. Machine Learning Models

In this study, common machine learning methods, including K-nearest neighbor (KNN), naive Bayes, random forest, decision tree, multilayer perceptron (MLP), and support vector machine (SVM), were used in conjunction with the traditional statistical method and logistic regression. Because it has previously been reported that nested cross-validation yielded better predictive accuracy than k-fold cross-validation [22, 23], in the present study, nested 5-fold cross-validation was used in machine learning. In nested cross-validation, an outer k-fold cross-validation loop is used to provide a performance estimate for the optimal model, and in each outer fold, an inner cross-validation is used to tune the parameters of the model [23]. A parameter set maximizing the area under the receiver operating characteristic curves (AUCs) was chosen, and the algorithm was trained using the training set with this parameter set.

In each model, AUC, sensitivity, specificity, *F*1-score, balanced classification rate, and accuracy were measured, and the performance of the model was compared based on the AUC. The performance measures are shown in Table 1 [24]. For a screening tool, sensitivity is more important than specificity to achieve timely diagnosis and treatment unless the specificity is very limited [25]. Because the ultimate purpose of the prediction models was screening people for an increased risk of MetS, sensitivity was used as a potentially distinguishing parameter of prediction models that exhibited similar performances.

To investigate the influence of incorporating Sasang constitution type into machine learning-based prediction of MetS, two models were chosen and the results from each one, with and without Sasang constitution factor type, were compared. Furthermore, potentially informative factors with regard to MetS prediction were investigated using the random forest method with reference to each Sasang constitution type to check whether the informative factors would be different by constitution type. All machine learning and statistical analysis procedures were performed using *R* software version 3.3.1 (R Foundation for Statistical Computing, Vienna, Austria). AUCs were used to compare the performances of the statistical methods and the machine learning methods. In statistical analyses, *p* < 0.05 was considered significant.

## 3. Results

### 3.1. Study Population Characteristics

Among 3,064 participants, 183 were excluded from the analysis due to missing data. Because the primary aim of the present study was to investigate the prediction of MetS via methods incorporating Sasang constitution type, the TY type was excluded from the analysis because that group only contained 10 individuals. Thus, a total of 2,871 participants were included in the final analysis, and 750 were classified as having MetS.

In the MetS group, 37.1% of the subjects were men, compared with 24.2% in the non-MetS group (*p* < 0.001). The mean age was significantly higher in the MetS group (0.54 ± 0.18) than in the non-MetS group (0.43 ± 0.18) (*p* < 0.001). The proportion of participants who had not graduated beyond middle school was higher in the MetS group (50.3%) than in the non-MetS group (35.5%). Compared with the non-MetS group, in the MetS group, the proportion of current smokers was significantly higher (*p* < 0.001), as were mean BMI (*p* < 0.001), alcohol consumption (*p*=0.003), and stress level (*p*=0.03). With regard to Sasang constitution, 68.9% of the individuals in the MetS group were classified as TE, compared with 38.9% in the non-MetS group (*p* < 0.001; Table 2).

### 3.2. Overall Comparison of the Machine Learning Models

The performances of the machine learning models are summarized in Table 3. Machine learning methods yielded AUCs of 0.73–0.80. Both MLP and SVM yielded the highest AUC of 0.80, as did logistic regression. The specificity of all models was ranged from 0.87 to 0.93. The sensitivity of MLP was substantially higher than that of SVM, but of all the machine learning-based methods investigated in this study, the naive-Bayes method yielded the highest sensitivity of 0.49 (Table 3).

### 3.3. Performances with and without Sasang Constitution Type

The incorporation of Sasang constitution type improved the sensitivity of models derived via all machine learning-based methods except for KNN. Notably, with the incorporation of Sasang constitution type, the sensitivity of the naive-Bayes-derived model improved from 0.40 to 0.49 (Table 3).

### 3.4. Factors to Predict MetS in Each Constitution Type

Table 4 shows the informative factors used to predict MetS for each Sasang constitution type. Without considering constitution type, BMI was the most informative predictor of MetS, followed by age and stress. These three parameters were also predictive in each Sasang constitution type. In subjects classified as TE type, physical activity and alcohol consumption contributed to MetS prediction to similar degrees, with respective values of 28.1 and 26.2. The same was true of SY type, with respective values of 35.0 and 35.8. With regard to the SE type, physical activity was more important than alcohol consumption (25.2 vs. 18.9). While BMI and age were significant predictors for all Sasang constitution types, there were differences in the relative significance of other factors between the constitution types (Table 4).

## 4. Discussion

In the current study, the performances of MLP and SVM were similar to that of conventional logistic regression modeling, and their AUC values were concordant with some previous reports [26, 27]. In one study using a conventional model, the AUC of MetS prediction using routine biomarkers was 0.796 in men and 0.897 in women [27]. In another study conducted in Taiwan, a risk prediction model for MetS yielded an AUC of 0.83 in subjects aged 35–74 years [26]. In a Korean study involving a 14-year longitudinal prospective cohort, the AUC of a prediction model was 0.81 without the incorporation of Sasang type and 0.82 with the incorporation of Sasang type [13]. Studies reporting that the performances of prediction models derived via machine learning methods can be similar to those of conventional statistical methods suggest that machine learning methods are a useful tool for MetS prediction.

In comparison with previously reported machine learning-derived models for MetS prediction, the models developed in the present study exhibited lower sensitivity (0.31–0.49), higher specificity (0.87–0.93), and a similar level of accuracy (0.73–0.80). In previous studies, MetS prediction models derived via machine learning have exhibited sensitivities ranging from 0.38 to 0.77, specificities ranging from 0.72 to 0.74, and accuracies ranging from 0.71 to 0.82 [8, 9]. In one MetS prediction study that utilized genetic and clinical information derived from a nonobese population, sensitivity was 0.38–0.42 and accuracy was 0.71–0.82 [8]. In another study investigating the prediction of MetS using decision tree and SVM methods, sensitivity ranged from 0.77 to 0.76, specificity ranged from 0.74 to 0.72, and accuracy ranged from 0.76 to 0.74 [9]. In Steinberg et al.'s [28] study using a big data analytic predictive platform, the AUC of a MetS prediction model was 0.8.

Differences in machine learning performance can be caused by various factors related to participants and analysis methods. Leeflang et al. [29] reported that disease prevalence affected the sensitivity and specificity of a test and that a higher disease prevalence was associated with a lower specificity. Previous studies have suggested that other factors that can affect the performance of prediction models, including imbalanced ratios in sampling techniques [30] and average fold change [31].

Incorporating Sasang constitution type may improve the performances of prediction models designed to screen for high-risk individuals, even though Sasang constitution type did not show higher importance compared to any other predictors. Like previous studies of MetS prediction showing small differences among models [8, 9], the differences between models with and without Sasang constitution type were not large in this study. However, in the present study, all of the prediction models investigated exhibited the same or higher AUCs with the incorporation of Sasang constitution type except for the decision tree. Furthermore, the incorporation of Sasang constitution type improved the sensitivity of the conventional regression method and all of the machine learning models except for KNN. In a previous machine learning study, constitution type was a significant predictor of MetS, as were total bilirubin and low-density lipoprotein cholesterol [32]. Increased performance was also indicated in a previous study in which Lee et al. [13] used statistical analysis to investigate a MetS prediction model and reported that the AUC increased significantly after the incorporation of Sasang constitution type. Sasang constitution type needs to be considered in the development of MetS prediction models in future studies.

It has previously been reported that MetS risk factors differ by Sasang constitution type [13, 33]. One study reported that age and BMI were significant MetS risk factors in TE and SY type individuals, but not in SE type individuals [13]. In another study, the best predictors of MetS risk in female participants were BMI in TE individuals and waist circumference in non-TE individuals, whereas in male participants, the best predictor was waist circumference in both TE and non-TE individuals [33]. In the present study, the three most influential MetS risk factors were BMI, age, and stress in individuals of all Sasang constitution types. Notably, while alcohol consumption was a more influential MetS predictor than physical activity in SY individuals, the importance of those two factors was similar in TE and SY individuals. There is a need for further studies investigating MetS risk factors in conjunction with Sasang constitution types to facilitate the development of MetS risk management guidelines.

The current study had several limitations. It did not include genetic factors, which may enhance the performance of prediction models. Previous studies have described genetic risk factors for MetS [34, 35], and in Cho et al.'s [8] study investigating the development of MetS prediction models via machine learning, a model using clinical and genetic data exhibited higher performance than a model using only clinical data. Future studies need to consider genetic factors in conjunction with Sasang constitution type in the development of MetS prediction models. Another limitation of the present study was that it was restricted to cross-sectional data. While many previous studies have assessed associations between risk factors and MetS using cross-sectional data [36, 37], cross-sectional studies have inherent limitations, including the order of disease occurrence and risk factors.

The current study is the first to generate prediction models to investigate the effects of Sasang constitution types on MetS via machine learning methods. Machine learning-derived models exhibited performances that were similar to that of conventional analysis with respect to MetS prediction, and the best algorithms for that purpose were identified among a panel of machine learning algorithms.

## 5. Conclusion

Of the six machine learning methods investigated in the present study, MLP and SVM exhibited MetS prediction performances that were similar to that of conventional statistical analysis. Notably, MLP and naive Bayes exhibited higher sensitivity than conventional statistical analysis. Given that the sensitivity of some models was increased via the incorporation of Sasang constitution type, Sasang constitution type needs to be considered in MetS prediction models.

## Figures and Tables

**Table 1:** Performance measures.

Measures	Description
Accuracy	*TP*+*TN*/*TP*+*FN*+*FP*+*TN*

Sensitivity	*TP*/*TP*+*FN*

Specificity	*TN*/*FP*+*TN*

Balanced classification rate	1/2(*TP*/(*TP*+*FN*)+*TN*/(*TN*+*FP*))

*F*1-score	2*TP*/2*TP*+*FP*+*FN*

FN, false negative; FP, false positive; TN, true negative; TP, true positive.

**Table 2:** Characteristics of participants with and without metabolic syndrome.

		No metabolic syndrome	Metabolic syndrome	Total	*p* value
Number (percentage)		2,121 (73.9)	750 (26.1)	2,871 (100.0)	
Sex	Male	513 (24.2)	278 (37.1)	791 (27.6)	<0.001
	Female	1,608 (75.8)	472 (62.9)	2,080 (72.4)	

Age^*∗*^		51.93 ± 7.18	56.19 ± 7.06	53.04 ± 7.39	<0.001
Education	Graduated middle school or less	753 (35.5)	377 (50.3)	1,130 (39.4)	<0.001
	Graduated from high school	818 (38.6)	218 (29.1)	1,036 (36.1)	
	Graduated college or higher	550 (25.9)	155 (20.7)	705 (24.6)	

Marriage status	Never married	48 (2.3)	13 (1.7)	61 (2.1)	0.058
	Married/living together	1,943 (91.6)	673 (89.7)	2,616 (91.1)	
	Separated/divorced/widowed	130 (6.1)	64 (8.5)	194 (6.8)	

Smoking	Never	1,762 (83.1)	551 (73.5)	2,313 (80.6)	<0.001
	Ex-smoker	187 (8.8)	113 (15.1)	300 (10.4)	
	Current	172 (8.1)	86 (11.5)	258 (9.0)	

BMI (kg/m^2^)^*∗*^		23.37 ± 2.47	26.03 ± 2.72	24.06 ± 2.80	<0.001
Alcohol consumption (g/day)^*∗*^		5.35 ± 17.44	8.01 ± 22.37	6.04 ± 18.89	0.003
Physical activity (MET minutes/week)^*∗*^		165.35 ± 217.03	163.20 ± 201.35	164.79 ± 213.01	0.81
Stress^*∗*^		19.49 ± 8.18	20.25 ± 8.32	19.68 ± 8.23	0.031

Sasang constitution	SY	632 (29.8)	163 (21.7)	795 (27.7)	<0.001
	TE	825 (38.9)	517 (68.9)	1,342 (46.7)	
	SE	664 (31.3)	70 (9.3)	734 (25.6)	

^*∗*^Mean ± standard deviation with *t*-test *p* value; other values are numbers followed by percentages in parentheses and analyzed by the chi-square test. BMI, body mass index; SE, So-Eum; SY, So-Yang; TE, Tae-Eum.

**Table 3:** Comparison of model performance between the model with and without Sasang constitution.

		AUC	Sensitivity	Specificity	*F*1-score	Balanced classification rate	Accuracy
KNN	Without SC	0.73	0.31	0.91	0.39	0.61	0.75
	With SC	0.73	0.31	0.91	0.40	0.61	0.75

Naive Bayes	Without SC	0.79	0.40	0.91	0.48	0.65	0.78
	With SC	0.79	0.49	0.87	0.53	0.68	0.77

Random forest	Without SC	0.77	0.36	0.92	0.45	0.64	0.78
	With SC	0.78	0.37	0.92	0.46	0.64	0.77

Decision tree	Without SC	0.78	0.35	0.93	0.45	0.64	0.78
	With SC	0.77	0.39	0.92	0.47	0.65	0.78

MLP	Without SC	0.8	0.45	0.91	0.52	0.68	0.79
	With SC	0.8	0.47	0.91	0.53	0.69	0.79

SVM	Without SC	0.8	0.37	0.93	0.48	0.65	0.79
	With SC	0.8	0.38	0.93	0.48	0.65	0.79

Logistic regression	Without SC	0.8	0.39	0.93	0.49	0.66	0.79
	With SC	0.8	0.40	0.93	0.49	0.66	0.79

Sex, age, education, marriage status, smoking, body mass index, alcohol, activity, and stress were included. AUC, area under the receiver operating characteristic curve; KNN, K-nearest neighbor; MLP, multilayer perceptron; SC, Sasang constitution; SVM, support vector machine.

**Table 4:** Order of informative value of variables associated with metabolic syndrome as identified by the random forest method.

Total		Sasang constitution					
		TE (*n* = 1,342)		SY (*n* = 795)		SE (*n* = 734)	
Variable	Importance	Variables	Importance	Variables	Importance	Variables	Importance
BMI^*∗*^	100.0	BMI^*∗*^	100.0	BMI^*∗*^	100.0	BMI^*∗*^	100.0
Age^*∗*^	49.4	Age^*∗*^	58.0	Age^*∗*^	64.6	Age^*∗*^	43.5
Stress^*∗*^	37.4	Stress^†^	43.8	Stress	53.7	Stress	42.6
Activity^*∗*^	24.2	Activity^†^	28.1	Alcohol consumption	35.8	Activity	25.2
Alcohol consumption	23.2	Alcohol consumption	26.2	Activity	35.0	Alcohol consumption	18.9
Sasang constitution^†^	16.0	Education^*∗*^	9.7	Education	13.1	Education^*∗*^	10.2
Education^*∗*^	8.7	Smoking^†^	4.7	Smoking	7.4	Marital status^†^	4.2
Smoking^*∗*^	3.8	Sex	1.6	Sex	2.3	Smoking	2.2
Sex^†^	1.1	Marital status	0.0	Marital status	0.0	Sex	0.01
Marital status	0.0	—	—	—	—	—	

^*∗*^ *p* < 0.05and ^†^*p* < 0.1 in logistic regression. BMI, body mass index; SE, So-Eum; SY, So-Yang; TE, Tae-Eum.

## Data Availability

The datasets used and/or analyzed during the current study are available from the National Institute of Health, Korea Disease Control and Prevention Agency, Republic of Korea, on reasonable request.

## Additional Points

In the prediction of metabolic syndrome, MLP and SVM exhibited the same performance as a regression method as determined via AUCs. Machine learning may be a useful tool for predicting metabolic syndrome. MLP and SVM exhibited the same AUCs as regression, but MLP exhibited higher sensitivity (0.47) than regression (0.40). Incorporation of Sasang constitution type improved the metabolic syndrome prediction sensitivity of all of the machine learning methods investigated except for KNN.

## Supplementary Materials

Supplementary Materials
*Supplementary 1*. Questionnaire for the Sasang constitution classification.
