# Supplementary material for: Metabolic Syndrome Prediction Models Using Machine Learning and Sasang Constitution Type
Source: Evid Based Complement Alternat Med. 2021 Feb 8;2021:8315047. doi: 10.1155/2021/8315047 (PMC7886522; doi:10.1155/2021/8315047)
Supplement: Supplementary Materials — Supplementary 1. Questionnaire for the Sasang constitution classification. [file 8315047.f1.docx]

Supplementary 1. Questionnaire for the Sasang constitution classification.

| ■ [QSCCII+] The following questions are for characterizing your Sasang Constitution.  ※ For each question, please mark the response that applies to you.  1) How would you describe your build?   ① Large  ② Average   ③ Small  2) How would you describe your body shape?   ① Chubby  ② Average  ③ Skinny  3) How would you describe the relation between your upper body and lower body?   ① My upper body has a stronger build than my lower body.  ② My lower body has a stronger build than my upper body.  ③ The upper and lower parts of my body are similar in build.  4) Which one of the following statements below best describes your body shape?   ① My neck area is strong, but my waist area is rather weak.  ② My chest area is strong, but my hip area is rather weak.  ③ My waist area is strong, but my neck area is rather weak.  ④ My hip area is strong, but my chest area is rather weak.  5) Which one of the following items below describes best your overall appearance and frame?   ① Chubby with a thick-set frame  ② Balanced with a small frame  ③ Average with a sturdy frame  6) How would you describe your chest area?   ① Broad and strongly built (obese type)  ② Frail and curved (leptosomatic type)  ③ Broad and sturdy (muscular type)  ④ Wide and balanced  7) Which type of food do you prefer?   ① Hot food  ② Cold food  8) Which one of the following statements below describes you best?   ① My hands and feet are usually cold  ② My hands and feet are usually warm  9) Which one of the following statements describes your gait?   ① My gait is slow and heavy.  ② My gait is natural and gentle.  ③ My gait is fast and my body sways while walking.  ④ My gait is straight and firm.  10) Which one of the following statements describes you best?   ① I tend to sweat much, and I feel refreshed when I sweat.  ② I tend not to sweat much, and I feel tired when I sweat even a little.  ③ I don’t feel tired when I sweat.  11) Which one of the following statements describes you best?   ① Carefree and prompt in action  ② Active and courageous  ③ Calm on the outside, while acting rather formally.  ④ Sweet and gentle, without pretension.  12) Which one of the following statements describes you best? (task performance or lifestyle)   ① I would go ahead with a given task without hesitation until completion.  ② I would go ahead with a new task but tend not to complete the task.  ③ I tend to work towards my own goals without unnecessary actions.  ④ I tend to stay in one place rather than visit places.  ※ Please tick the box (√) next to the statements that seem to match your general feelings and behaviors.   13) I complete a given task without hesitation or reservation. □  14) I am active, but rather stubborn. □  15) I tend to take action without contemplating the gain or loss. □  16) I am able to stay in one place for a long time. □  17) I am careful and meticulous. □  18) I tend to associate with people close to me. □  19) I am able to make friends easily without judging them. □  20) I am more focused on my personal tasks than on social tasks. □  21) I tend to value external things more than internal things. □  22) I find it difficult to make friends as I often think too much. □  23) I am tidy and discrete. □  24) I am carefree and open. □  25) I have a sweet temper and I am able to soothe people well. □  26) I like participating in various social activities. □  27) I am able to tell at a glance whether a person is diligent or not. □  28) I always hurry and rush myself. □  29) I find it difficult to speak or perform in front of others at first. □  30) I am timid and feel constantly anxious. □  31) I am usually sensitive. □  32) I am patient. □  33) I tend to act rashly. □  34) I feel nervous and anxious even for things that are no big deal. □  35) I have more masculine characteristics than feminine ones. □  36) I feel annoyed even for things that are not important. □  37) I find it difficult to speak or give presentations in front of others. □  38) I am usually cheerful, but I frequently experience mood swings. □  39) I tend to hide my feelings when I am hurt emotionally. □  40) I am unable to control my anger when someone humiliates me. □  41) I tend not to speak up until I am certain that my thoughts are right. □  42) I frequently pretend to know things or show off to people. □  43) I am polite and respectful towards others but not to myself. □  44) I find it difficult to stand in front of people. □  45) I tend to act carefree without thinking about what others might think. □  46) I am timid and feel constantly anxious. □  47) I have more feminine characteristics than masculine characteristics. □  48) I feel inadequate even when a significant task has been completed. □  49) I have experienced vomiting or difficulty in swallowing without any cause. □  50) I feel refreshed after sweating when I am ill. □  51) I have indigestion when I am ill. □  52) I sigh a lot when I feel tired performing my daily activities. □  53) I have experienced feeling listless and finding it difficult to move. □  54) I frequently feel that my stomach and chest are heavy after eating food. □ |
| --- |
